# Angiotensin Converting Enzyme Inhibitor and HMG-CoA Reductase Inhibitor as Adjunct Treatment for Persons with HIV Infection: *A Feasibility Randomized Trial*

**DOI:** 10.1371/journal.pone.0046894

**Published:** 2012-10-17

**Authors:** Jason V. Baker, Kathleen Huppler Hullsiek, Rachel Prosser, Daniel Duprez, Richard Grimm, Russell P. Tracy, Frank Rhame, Keith Henry, James D. Neaton

**Affiliations:** 1 Department of Medicine, Hennepin County Medical Center (HCMC), Minneapolis, Minnesota, United States of America; 2 Department of Medicine, University of Minnesota, Minneapolis, Minnesota, United States of America; 3 Department of Biostatistics, University of Minnesota, Minneapolis, Minnesota, United States of America; 4 Department of Biochemistry, University of Vermont, Burlington, Vermont, United States of America; 5 Department of Medicine, Abbott Northwestern Hospital, Minneapolis, Minnesota, United States of America; Institut National de la Santé et de la Recherche Médicale, France

## Abstract

**Background:**

Treatments that reduce inflammation and cardiovascular disease (CVD) risk among individuals with HIV infection receiving effective antiretroviral therapy (ART) are needed.

**Design and Methods:**

We conducted a 2×2 factorial feasibility study of lisinopril (L) (10 mg daily) vs L-placebo in combination with pravastatin (P) (20 mg daily) vs P-placebo among participants receiving ART with undetectable HIV RNA levels, a Framingham 10 year risk score (FRS) ≥3%, and no indication for ACE-I or statin therapy. Tolerability and adherence were evaluated. Longitudinal mixed models assessed changes in blood pressure (BP), blood lipids, and inflammatory biomarkers from baseline through months 1 and 4.

**Results:**

Thirty-seven participants were randomized and 34 [lisinopril/pravastatin (n = 9), lisinopril/P-placebo (n = 8), L-placebo/pravastatin (n = 9), L-placebo/P-placebo (n = 8)] attended at least one follow-up visit. Participants were 97% male, 41% white, 67% were current smokers, and 65% were taking a protease inhibitor. Median age was 48 years, CD4 count 483 cells/mm^3^, FRS 7.79%, total cholesterol 184 mg/dL, and LDL-C 95 mg/dL. There was no treatment difference for pravastatin vs P-placebo in total cholesterol, LDL-C, or any of the inflammatory biomarkers. Participants randomized to lisinopril vs. L-placebo had significant declines in diastolic BP (−3.3 mmHg, p = 0.05), hsCRP (−0.61 µg/mL, p = 0.02) and TNF-α (−0.17 pg/mL, p = 0.04). Participants taking lisinopril vs L-placebo were more likely to report missed doses (88 vs 35%; p = 0.001) and have adherence <90% by pill count (42 vs. 0%; p = 0.02). Few participants from either group reported side effects (n = 3 vs. n = 1).

**Conclusions:**

The modest BP changes and decreased adherence with lisinopril and absence of lipid differences with pravastatin suggest future studies of these drug classes should consider a run-in period to assess adherence and use a different statin. Our results also indicate that ACE-I therapy may have anti-inflammatory benefits for ART-treated persons with HIV infection and this should be further evaluated.

**Trial Registration:**

ClinicalTrials.gov NCT00982189

## Introduction

Individuals with HIV infection are at increased risk for premature cardiovascular disease (CVD) due to the higher prevalence of traditional risk factors (e.g., smoking), toxicity from antiretroviral therapy (ART; e.g., metabolic complications), as well as direct effects of HIV itself [1]. Specifically, HIV-related inflammation persists despite effective viral suppression with ART treatment and this may further amplify CVD risk [2], [3], [4], [5]. CVD prevention strategies that encompass both anti-inflammatory benefits as well as traditional risk factor modification may be uniquely beneficial in this context.

Similar to the general population, high blood pressure (BP) and cholesterol account for a significant proportion of CVD risk among patients with HIV infection and remain a key component of prevention strategies [6]. In the general population, epidemiologic data demonstrate a consistent graded relationship between BP and cholesterol with CVD, which persists through normal BP values (down to at least 115/75 mmHg) and moderate total cholesterol levels (155 to >200 mg/dL) [7], [8]. For a target population at higher absolute CVD risk, such as individuals with HIV infection, these data suggest risk factor reductions may be beneficial irrespective of whether individual BP or cholesterol levels exceed current thresholds for treatment [9], [10].

Angiotensin converting enzyme inhibitors (ACE-I) and HMG-CoA reductase inhibitors (‘statins’) have been shown to reduce CVD risk through their BP and cholesterol lowering properties, respectively [11], [12]. However, both classes of medications appear to have additional anti-inflammatory pleotropic effects that may be uniquely beneficial for HIV positive patients [13], [14], [15]. Prior to expanding the use of ACE-I and/or statins for HIV-infected persons to patients for whom these treatments are not currently indicated, safety and tolerability data are needed to inform large-scale trials that more clearly define the net risk-benefit balance.

The goal of this study was to determine if a strategy using lisinopril (an ACE-I) at 10 mg daily and pravastatin (a ‘statin’) at 20 mg daily as adjunctive treatment was feasible, well tolerated, and led to risk factor reductions when given alone or in combination to virologically suppressed patients receiving ART. We also explored the potential treatment effect on biomarkers of systemic inflammation: high sensitivity C-reactive protein (hsCRP), interleukin-6 (IL-6) and tumor necrosis factor-alpha (TNF-α).

## Methods

The protocol for this trial and supporting CONSORT checklist are available as supporting information; see Checklist S1 and Protocol S1.

### Participants

Participants with HIV infection receiving ART with HIV RNA levels <200 copies/mL and a FRS ≥3% for 10-year coronary heart disease risk were enrolled after written informed consent at one of two HIV clinics (Hennepin County Medical Center [HCMC] and Clinic 42, Allina Hospitals and Clinics, Minneapolis, Minnesota) from January 2010 through February 2011. Exclusion criteria included known CVD, hypertension or BP ≥140/90 mmHg, low-density lipoprotein cholesterol (LDL-C) >160 mg/dL (or >130 mg/dL with a FRS >10%), triglycerides >500 mg/dL, diabetes, cirrhosis, serum creatinine ≥2.0 mg/dL, or a contra-indication to taking ACE-I or statin therapy. Our criteria specifying a FRS ≥3% both eliminates those at very low risk for CVD and efficiently selects for a target population at moderate CVD risk but for whom BP or cholesterol lowering therapy were not typically indicated. When applying data that HIV infection is associated with approximately a 2-fold increased CVD risk, our target population should have at least a 5% risk for coronary event in next 10 years [16]. FRS used for analyses was calculated from the published algorithm that considered age, gender, systolic BP, total cholesterol, high-density lipoprotein [HDL-C], and current smoking and diabetes status [17]. For screening purposes, FRS was estimated through application of a point-of-care online calculator (hp2010.nhlbihin.net/atpiii/calculator.asp). The study was approved by the institutional review board at each clinical site (HCMC Human Subjects Research Committee and Allina Hospitals and Clinics Institutional Review Board) and the protocol was registered at ClinicalTrials.gov (NCT00982189).

### Study Design

**Figure 1:**
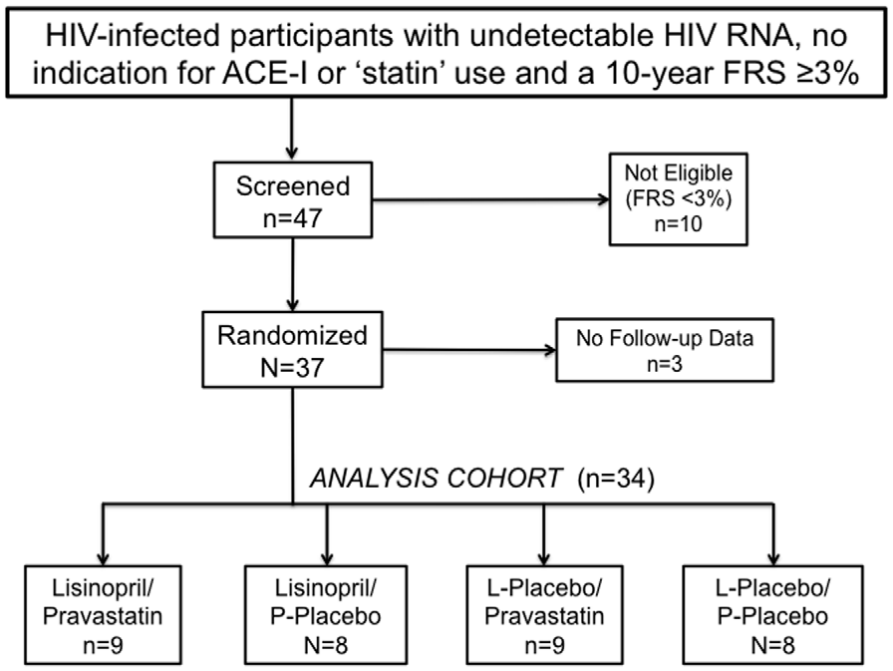
Study Design Flow-Diagram.

The study design was a randomized, double-blinded 2×2 factorial design of lisinopril (L) 10 mg daily versus matched L-placebo daily in combination with pravastatin (P) 20 mg daily versus P-placebo (**Figure 1**). The goal of the study was to inform planning for larger studies by first assessing ability to recruit for the proposed interventions, and assessing tolerability and adherence. Planned sample size was 40 participants, which provided 80% power to detect a 12.6 mmHg difference between the lisinopril and L-placebo groups for systolic BP (assuming a standard deviation of 14 mmHG) and a 27 mg/dL difference in LDL cholesterol between the pravastatin and P-placebo groups (assuming a standard deviation of 30 mg/dL). The sample size and recruitment period was limited by budget and time restrictions associated with the American Heart Association funding mechanism supporting the study.

In order to blind both participants and study investigators to the treatment assigned, active study drugs were over-encapsulated to match the respective placebo capsules. Treatment allocation was balanced in blocks of 4 or 8 providing four groups of approximately equal size. Treatment schedules were only known to the unblinded statistician and the pharmacist responsible for preparing study medication bottles; neither had contact with study participants. After the baseline assessment, participants were instructed to take 1 capsule from each study medication bottle by mouth daily and returned for repeat study visit procedures at 1 and 4 months.

### Outcomes

Adherence was assessed via participant self-reported estimates of number of missed doses per week during the study, and then objectively at month 4 by pill count. Tolerability and safety were ascertained through participant history at both 1 and 4 month visits. A fasting lipid profile (total cholesterol, LDL-C, HDL-C, and triglycerides) was obtained at the site clinical laboratory at baseline, 1 and 4 months. Research nurses measured BP in triplicate at each study visit, with mean values used for analyses. Plasma biomarkers of systemic inflammation were also assessed at baseline and the 1 and 4-month follow-up visits. Inflammatory markers were measured by the Laboratory for Clinical Biochemistry Research at the University of Vermont; hsCRP was measured with a NB™II nephelometer, N Antiserum to Human CRP (Siemens Diagnostics, Norwood, MA); IL-6 with Chemiluminescent Sandwich ELISA (R&D Systems, Minneapolis, MN); and TNF-α with Millipore Panel B multiplex (Billerica, MA). The lower level of detection for hsCRP, IL-6, and TNF-α were 0.16 µg/mL, 0.16 pg/mL, and 0.32 pg/mL. All samples were analyzed blinded to treatment group. The assay coefficient of variance (CV) using these methods is 5% for hsCRP, 7% for IL-6, and 8% for TNF-α. In addition to these measures we also obtained a basic metabolic panel, aspartate aminotransferase, alinine aminotransferase, serum creatine kinase, and a complete blood count, and HIV clinical labs (HIV RNA level and CD4+ T-cell count) at each visit.

### Statistical Methods

All analyses are based on the participants who had at least one follow-up visit. Descriptive statistics were calculated to compare self-reported side-effects and medication adherence and pill counts for each treatment (lisonopril versus L-placebo and pravastatin versus P-placebo); p-values for those comparisons are from Fisher's exact tests. A missed follow-up visit was assigned the worse adherence category (>3 misses per week). Longitudinal mixed effects regression models which used all available follow-up data were used to assess treatment effects on change in BP, lipids, and inflammatory biomarkers averaged over both month 1 and 4 visits. Models included the baseline level of the outcome of interest, main effects for lisinopril and pravastatin, and the interaction between those treatments. None of the interaction terms were significant and therefore they were removed from the final models; only the results of main effects models are presented. Levels of hsCRP, IL-6 and TNF-α were log_e_–transformed before analysis; results are reported on the original scale after back-transformation. A global assessment measure described by O'Brien was also considered for the inflammatory biomarkers since we expected the treatments to have a similar effect on all of markers [18]. With this “rank-sum” method the values of hsCRP, IL-6 and TNF-α were ranked and then summed for each treatment group. Models as described above were used to compare the treatments for sum of the ranks. The comparisons of the inflammatory markers are considered exploratory. Two-sided p-values and 95% confidence intervals (CIs) are cited, with no adjustments for multiple comparisons. Analyses were performed using SAS version 9.2 (SAS Institute) and R version 2.9.

## Results

### Study Population

Forty-seven participants completed a screening visit and 37 of these were randomized (**Figure 1**). The reason for screening failure (n = 10) was a FRS <3%. Three persons who were randomized withdrew consent prior to starting study medication and had no follow-up data (2 allocated to lisinopril/P-placebo group and 1 allocated to L-placebo/P-placebo group). Of the remaining 34 participants in the final analysis cohort, one missed the month 1 visit (from L-placebo/P-placebo group) and one missed the month 4 visit (from Lisinopril/P-placebo group). Baseline characteristics for the study population are presented in **Table 1**. Median age was 48, only one female was randomized, the majority (68%) reported smoking cigarettes, median CD4 count was 482 cells/mm^3^, and most (65%) participants were taking protease-inhibitor-based ART. No participants had a prior history of injection drug use. Median FRS was 7.9%, and BP and cholesterol levels were below treatment thresholds for persons without prior CVD. There were no significant differences in baseline characteristics between lisinopril and L-placebo groups, pravastatin and P-placebo groups, or when compared across all 4 of the individual treatment combinations.

**Table 1:** Baseline Characteristics.

	Overall	Lisinopril	L-placebo	Pravastatin	P-placebo
**Totals (N)**	**34**	**17**	**17**	**18**	**16**
Age (years), median [IQR]	48 [44, 56]	49 [45, 53]	47 [44, 56]	48 [45, 56]	51 [44, 55]
Male gender (%)	33 (97%)	17 (100%)	16 (94%)	17 (94%)	16 (100%)
Race/ethnicity					
White (%)	14 (41%)	5 (29%)	9 (53%)	7 (39%)	7 (44%)
African Am (%)	16 (47%)	11 (65%)	5 (29%)	9 (50%)	7 (44%)
Hispanic (%)	3 (9%)	0	3 (18%)	1 (6%)	2 (13%)
Other (%)	1 (3%)	1 (6%)	0	1 (6%)	0
IDU, never (%)	34 (100%)	17 (100%)	17 (100%)	18 (100%)	16 (100%)
Smoker (%)	23 (68%)	13 (76%)	10 (59%)	11 (61%)	12 (75%)
Hepatitis B or C (%)	11 (33%)	4 (24%)	7 (41%)	4 (22%)	7 (44%)
ART Regimen, PI-based (%)	22 (65%)	8 (47%)	14 (82%)	11 (61%)	11 (69%)
CD4 cells/mm^3^,median [IQR]	483 [310, 609]	429 [320, 595]	522 [288, 609]	417 [288, 535]	579 [367, 708]
**CVD RISK FACTORS**					
SBP (mmHg), median [IQR]	123 [117, 126]	122 [118, 125]	123 [117, 128]	124 [117, 128]	122 [114, 126]
DBP (mmHg), median [IQR]	76 [70, 79]	76 [71, 77]	77 [70, 83]	77 [70, 79]	74 [70, 80]
T. Chol (mg/dL), median [IQR]	184 [162, 196]	182 [162, 196]	185 [165, 195]	187 [165, 195]	179 [158, 197]
LDL-C (mg/dL), median [IQR]	95 [86, 115]	93 [78, 110]	98 [91, 119]	96 [86, 119]	95 [85, 111]
HDL-C (mg/dL), median [IQR]	42 [35, 54]	51 [38, 60]	35 [35, 49]	43 [35, 53]	42 [35, 76]
FRS 10-year, median [IQR]	7.9 [4.7, 10.6]	7.6 [6.5, 10.6]	8.0 [4.5, 10.2]	7.9 [5.9, 9.4]	7.8 [4.5, 11.0]
**BIOMARKERS**					
hsCRP (µg/mL), median [IQR]	1.37[0.55, 2.52]	1.34[0.56, 2.25]	1.40[0.55, 2.52]	1.74[0.56, 2.93]	1.30[0.44, 1.97]
IL-6 (pg/mL), median [IQR]	1.47[0.95, 2.31]	1.57[0.95, 2.82]	1.37[0.95, 2.17]	1.37[1.2, 2.31]	1.60[0.90, 2.49]
TNF-α (pg/mL), median [IQR]	3.99[2.99, 4.62]	4.51[3.81, 4.81]	3.72[2.99, 4.17]	4.13[2.99, 4.99]	3.85[2.94, 4.53]

### Tolerability and Adherence

Few participants reported side effects to study medication during the 4-month study period (**Table 2**). One participant each in the lisinopril and L-placebo groups reported cough. The participant receiving active lisinopril was unblinded and stopped the study medication. Additional specific side effects reported include nausea (n = 1) and runny nose (n = 1). Despite no difference in self-reported side effects, participants taking lisinopril vs. L-placebo reported lower rates of perfect adherence (i.e., no missed doses) at month 1 (59% vs. 100%; p = 0.007) and month 4 (13% vs. 65%; p = 0.007). Of the participants who returned study medication (n = 25), adherence of >90% of possible doses (assessed by pill count) was achieved by 58% of those randomized to lisinopril and 100% of those taking L-placebo (p = 0.01). Of participants who did not return study medications at month 4, one discarded his remaining supply and the others failed to return the bottles after repeated requests by the study coordinator. Side effects and adherence (assessed by self report or via pill count) did not differ between pravastatin and P-placebo groups (**Table 2**).

**Table 2:** Toxicity and Adherence During Follow-up.

	Lisinopril	L-Placebo	p-value^1^
**Side Effects (anytime during study)**	18% (3)	6% (1)	0.60
Cough	6% (1)	6% (1)	
**Missed Doses/Week (self-report at end of study)**			0.001
None	12% (2)	65% (11)	
1–2 misses/week	24% (4)	24% (4)	
≥3 or declined to specify	65% (11)	12% (2)	
**>90% Adherence** (Pill count at end of study; data available for n = 12 in Lisinopril group and n = 13 in L-Placebo group)	58% (7)	100% (13)	0.01

	Pravastatin	P-Placebo	p-value
Side Effects (anytime during study)	11% (2)	13% (2)	>0.99
**Missed Doses/Week (self-report at end of study)**			>0.99
None	39% (7)	38% (6)	
1–2 misses/week	22% (4)	25% (4)	
≥3 or declined to specify	39% (7)	38% (6)	
**>90% Adherence** (Pill count at end of study; data available for n = 15 in Pravastatin group and n = 10 in P-Placebo group)	80% (12)	80% (8)	>0.99

1 *P-values from Fisher's exact tests; missed follow-up visit was assigned ‘declined to specify’ for adherence; n = 34 was used for analysis of self-reported side effects and missed doses; pill-count adherence was assessed for sample of n = 25 with data available as indicated.*

### Treatment Effect of Lisinopril on Blood Pressure

**Figure 2:**
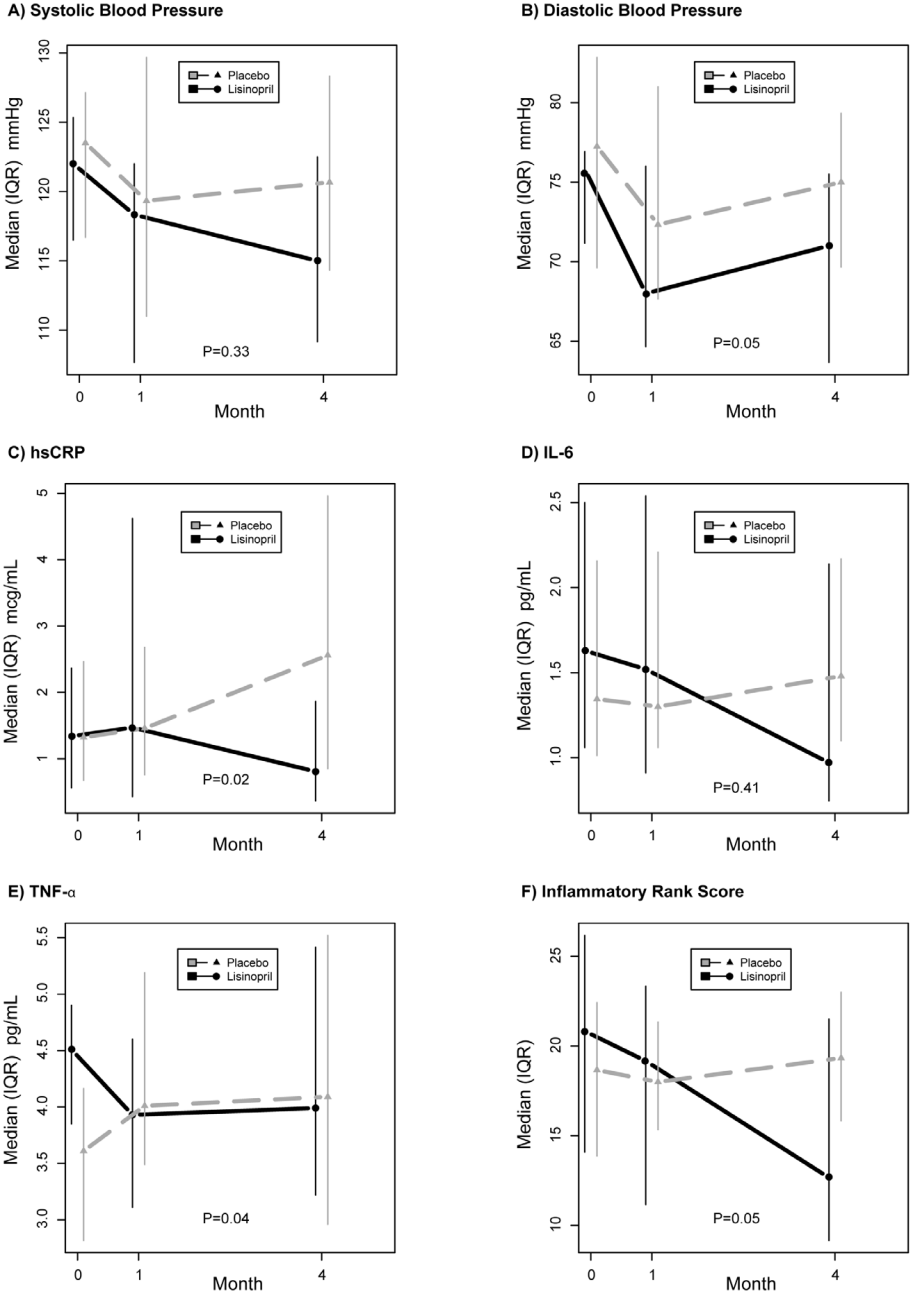
Median (IQR) Levels of Blood Pressure and Biomarkers of Inflammation for Lisinopril versus L-placebo Treatment Groups. Values are plotted at baseline and month 1 and 4 for: a) systolic blood pressure, b) diastolic blood pressure, c) hsCRP level, d) IL-6 level, e) TNF- α level, and f) the inflammatory rank score (the rank sum for hsCRP, IL-6 and TNF-α levels). Lisinopril group is in black (solid line) and L-placebo in grey (dashed line). P-values represent treatment comparisons from longitudinal models that estimate the average differences between groups over follow-up after adjusting for baseline value (see text for absolute estimates).

Compared to L-placebo, declines in systolic and diastolic BP were greater at both 1 and 4 months and averaged across both visits. For the latter, the treatment difference averaged across follow-up was −2.6 mmHg (95% CI −8.1, 2.8; p = 0.33) for systolic BP and −3.3 mmHg (95% CI −6.5, −0.1; p = 0.05) for diastolic BP (**Table 3** **and** **Figure 2**).

**Table 3:** The Treatment Effect of Lisinopril (n = 17) Versus L-Placebo (n = 17) on Blood Pressure.

	Month 1 Change^1^ (95% CI)	Month 4 Change^1^ (95% CI)	Average Change During Follow-up (95% CI)^2^	*p*-value^2^
Systolic BP (mmHg)	−3.4 (−8.9, 2.1)	−1.8 (−9.4, 5.8)	−2.63 (−8.1, 2.8)	0.33
Diastolic BP (mmHg)	−3.3 (−6.9, 0.2)	−3.3 (−7.3, 0.7)	−3.3 (−6.5, −0.1)	0.05

1 *) Regression models of treatment difference, adjusted for baseline level.*

2 *) Longitudinal mixed models of changes over both follow-up time points, adjusted for baseline level.*

### Treatment Effect of Pravastatin on Cholesterol

There were no significant differences in total cholesterol, LDL-C, triglycerides, HDL-C, or total-to-HDL-C ratio between pravastatin and P-placebo groups across follow-up visits (**Table 4**).

**Table 4:** The Treatment Effect of Pravastatin (n = 18) Versus P-Placebo (n = 16) on Blood Lipid Levels (n = 34).

	Month 1 Change^1^ (95% CI)	Month 4 Change^1^ (95% CI)	Average Change During Follow-up (95% CI)^2^	*p*-value^2^
Total Chol. (mg/dL)	0.08 (−12.42, 12.59)	−1.75 (−18.74, 15.25)	9.5 (−2.5, 21.5)	0.96
LDL-C (mg/dL)	−2.29 (−16,79, 12.22)	−0.62 (−13.22, 11.98)	5.5 (−6.2, 17.2)	0.87
HDL-C (mg/dL)	5.74 (−1.75, 13.24)	0.97 (−8.42, 10.36)	3.81 (−2.3, 10.0)	0.28
TC/HDL-C	−0.42 (−0.81, −0.04)	−0.07 (−1.11, 0.97)	0.01 (−0.6, 0.6)	0.39

1 *) Regression models of treatment difference, adjusted for baseline level.*

2 *) Longitudinal mixed models of changes over both follow-up time points, adjusted for baseline level.*

### Inflammatory Biomarkers

Baseline levels of hsCRP, IL-6 and TNF-α are reported (table 1), and there were no differences between treatment groups at study entry. At baseline, 13 (35%) participants had hsCRP levels categorized as low-risk for CVD (<1.0 mg/L), with the remaining either average- (n = 18, hsCRP 1.0–3.0 mg/L) or high- (n = 6, hsCRP >3.0 mg/L) CVD risk [19]. Figure 2 presents the median values during follow-up. The average change (decrease) from baseline during follow-up (estimated from longitudinal models) was greater for lisinopril compared to L-placebo for hsCRP (−0.54 µg/mL, 95% CI −0.90, −0.32; p = 0.02), IL-6 (−0.88 pg/mL, 95% CI −1.21, 0.64; p = 0.41), TNF-α (−0.84 pg/mL, 95% CI −0.99, −0.71; p = 0.04). These differences correspond to relative reductions for the lisinopril group of 42% for hsCRP and 23% for TNF-α. The treatment effect for hsCRP was not apparent until month 4 (−0.34 µg/mL; p = 0.02), whereas the decline in TNF-α was present by month 1 (−0.81 pg/mL; p = 0.02). Finally, the inflammatory score improved over follow-up for the group randomized to lisinopril vs. L-placebo (−2.81, 95% CI −5.68 to 0.05; p = 0.054).

There was no evidence of a pravastatin treatment effect on any of the inflammatory biomarkers at month 1, month 4, or averaged over both follow-up visits. There were also no differences in the lisinopril treatment effect on biomarker changes between those receiving or not receiving pravastatin (data not shown).

## Discussion

This feasibility study of lisinopril and/or pravastatin as a CVD prevention strategy for HIV positive persons taking effective ART with viral suppression provides important information for future trials directed at BP and lipid changes among HIV positive patients who do not have an indication for these drugs. We found that adherence to lisinopril (at 10 mg daily) was less than for matched L-placebo, and as a consequence blood pressure lowering was modest. Improvements in blood lipids were not evident with pravastatin (at 20 mg daily) though this effect may have been limited by the relatively low potency at this dose as well as the small sample size. Importantly, among these virologically suppressed patients, we also found that lisinopril led to short-term improvements in biomarkers of systemic inflammation (hsCRP and TNF-α).

Epidemiologic data suggests persons with HIV infection have an approximate 2-fold increased risk for CVD, when compared to the general population [16], [20], [21]. Whether absolute CVD event rates will continue to differ and widen further over time is unclear, given some evidence that more aggressive management of traditional risk factors in contemporary HIV care has attenuated CVD risk [22]. Newer antiretroviral medications may also continue to reduce metabolic complications. However, even with optimal management of BP and cholesterol to levels below clinical treatment thresholds based on risk, factors unique to HIV disease still appear to result in excess CVD events [4], [23], [24]. Furthermore, by focusing on extreme elevations in individual risk factors there are missed opportunities to reduce CVD risk through modest simultaneous reductions in multiple risk factors. This, combined with the observation that CVD risk can be reduced among persons without clinically overt CVD by lowering BP within normotensive values or lowering LDL-C beyond 130 mg/dL [25], [26], [27], [28], motivates the strategy of combining low-doses of various CVD prevention medications into a singly daily pill as prevention (i.e., the polypill) [9], [10], [29]. Our data provide some support that such a study is feasible in individuals with HIV infection, but, consistent with data from general population [30], [31] issues of tolerability, adherence, and potency will need to be carefully considered. Future studies like this should consider a run-in period to assess adherence or use of better-tolerated medications with similar mechanisms (e.g., angiotensin receptor blockers).

The proportion of ART-treated HIV infected patients in clinical practice that currently have no indication for treatment with an ACE-I or a statin likely varies widely by setting. The reported prevalence for dyslipidemia (e.g., cholesterol >200–240 mg/dL, HDL <35 mg/dL, receipt of lipid-lowering therapy or clinical diagnosis) has ranged from 30–35% [16], [22], [23], [24]. For hypertension (BP ≥140/90 mmHg, receiving BP lowering therapy or a clinical diagnosis), it is between 10–30%, and for a prior history of CVD between 5–10% [16], [22], [23], [24]. When one also excludes persons at very low risk CVD risk (e.g., unlikely to benefit from aggressive prevention efforts), the target population for a pre-emptive CVD prevention strategy will likely include much less than half of patients in most HIV clinical settings. Furthermore, CVD prevention treatments will likely be most effectively implemented if they target patients receiving ART whose risk for AIDS complications is low. Despite these potential exclusions, the difference between a low-burden of CVD risk factors and optimally managed risk factors still has substantial implications for longer-term CVD risk over a lifetime [32]. Defining the appropriate target population that optimizes the net benefit-risk balance will be an important goal for future HIV-related CVD prevention studies.

Inflammation is a key factor in the pathogenesis of cardiovascular disease and a hallmark of HIV infection that persists despite effective treatment with ART for years [2], [5]. The reasons for chronic immune activation and inflammation are multi-factorial, but potential drivers include residual low-level HIV replication, translocation of microbial products across damaged mucosal barriers, the presence of co-pathogens (e.g., herpes viruses or hepatitis B or C), as well as metabolic complications (e.g., increased visceral adiposity) [33], [34], [35], [36]. In this context, anti-inflammatory treatments are particularly attractive candidates for HIV-related CVD prevention, whether or not they target HIV-specific mechanisms or down-regulate inflammatory pathways more broadly. ACE-I and statins have been associated with anti-inflammatory effects [13], [14], [15]. We found that among persons with HIV infection, lisinopril use was associated with a decline in biomarkers of systemic inflammation. Favorable changes were evident in spite of suboptimal adherence. High-sensitivity CRP, specifically, is elevated with HIV infection and associated with risk for CVD among both HIV-infected and uninfected persons [2], [3], [20].

Our findings were limited by the small sample size. Confidence intervals are wide and we may have missed important treatment effects. Low power also limited our ability to detect treatment interactions. The lack of a treatment effect of pravastatin may be due to the low potency of this statin, as we did not detect changes in cholesterol or lipoproteins. Given the approach we were studying (i.e., adding pravastatin as primary prevention to asymptomatic patients) we chose a starting dose (e.g., 20 mg) to minimize risk/tolerability and our short-term follow-up duration precluded dose escalation. Other HIV studies using higher doses (i.e., pravastatin 40 mg daily) or other statins (e.g., atorvastatin and rosuvastatin) have demonstrated reductions in measures of immune activation or inflammation [13], [37].

In summary, our results support the feasibility of conducting further studies of similar adjunct treatments that may have multiple beneficial effects such as reducing BP and systemic inflammation among HIV positive patients. Adherence concerns with lisinopril in this context suggest other, more tolerable medications with similar effects on the renin-angiotensin-aldosterone-system (e.g., angiotensin receptor blockers), may be a more effective strategy. Ultimately, in addition to larger feasibility studies, HIV clinical outcome trials will have to performed to assess the risk/benefit of such adjunctive treatment strategies.

## Supporting Information

Checklist S1 **CONSORT Checklist.**
(DOC)

Protocol S1 **Trial Protocol.**
(PDF)
