# Supplementary material for: Angiotensin Converting Enzyme Inhibitor and HMG-CoA Reductase Inhibitor as Adjunct Treatment for Persons with HIV Infection: A Feasibility Randomized Trial
Source: PLoS One. 2012 Oct 17;7(10):e46894. doi: 10.1371/journal.pone.0046894 (PMC3474775; doi:10.1371/journal.pone.0046894)
Supplement: Protocol S1 — Trial Protocol. (PDF) [file pone.0046894.s002.pdf]

Cardiovascular Disease Risk Reduction for Persons with HIV Infection: *A 'polypill' pilot study*

[PCC-003]

ClinicalTrials.gov Identifier: NCT00982189

A Positive Care Center (PCC) single-site clinical trial conducted at HCMC

Funded by the American Heart Association

Oversight by Minneapolis Medical Research Foundation (MMRF)

Participating Clinical Centers:
Positive Care Center at Hennepin County Medical Center
Infectious Disease Clinic ('Clinic 42') at Abbott Northwestern Hospital (Allina)

Protocol Chair: Jason Baker, MD, MS

Protocol Team: W. Keith Henry, Rachel Prosser, Frank Rhame, Kathy Huppler-Hullsiek, Kristen Gamache, Bette Borndenave, Deborah Wood, Mark Olson, Daniel Duprez, Richard Grimm and James Neaton

Table of Contents

1. Background and Rationale **page 3**

- 1.1. Study Goal
- 1.2. Study Hypothesis
- 1.3. HIV/ART-related CVD Risk
- 1.4. Blood Pressure and Lipid Lowering Treatment to Reduce CVD Risk

2. Methodology **page 5**

- 2.1. Study Design
- 2.2. Outcomes
- 2.3. Design Justification
- 2.4. Data Collection
- 2.5. Sample Size Calculations
- 2.6. Study Procedures
- 2.7. Recruitment and Enrollment

3. Clinical Management **page 10**

- 3.1. Training and Administration of Study Procedures
- 3.2. Participant Safety Monitoring and Withdrawal

4. Analysis Plan **page 11**

5. References **page 12**

APPENDIX A – Consent Form **page 16**

1. BACKGROUND AND RATIONALE

Premature atherosclerotic cardiovascular disease (CVD) is now a leading cause of morbidity and mortality among persons with HIV-infection. In this population, premature CVD is a consequence of antiretroviral therapy (ART) toxicity, HIV itself, and a higher prevalence of traditional risk factors such as smoking.[1-13] To date, data are lacking to inform the use of well-tolerated adjunct primary CVD prevention strategies in persons with ART-treated HIV infection.

The positive linear relationship between CVD event risk and low-density lipoprotein cholesterol (LDLc) levels or systolic blood pressure has been well described within general population cohorts, and extends well below the thresholds for lipid or blood pressure (BP) lowering therapy, respectively.[14-16] Thus, treatment to lower BP and improve lipid profiles among those who would not otherwise receive it could have important health benefits in HIV-infected persons, where CVD risk is higher. In addition, 'statin' (HMG-CoA reductase inhibitor) drugs and angiotensin II converting enzxyme inhibitors (ACEi) also possess anti-inflammatory and anti-thrombotic properties that may also counter HIV/ART-related mechanisms that promote CVD.[17-21]

Safe treatments that target traditional CVD risk factors (BP and LDLc) in addition to HIV/ART-related vascular dysfunction, inflammation and thrombogenesis could then be combined to achieve simultaneous reductions in several CVD risk factors. Similar 'poly-pill' strategies have been studied in the general population, and would be highly effective at improving medical outcomes for the well over 1 million persons with HIV-infection in the U.S.—50% of whom will be over age 50 by 2015. The focus of this study is to provide pilot data that evaluates the safety and feasibility of a polypill CVD prevention strategy in persons with HIV infection.

1.1. Study Goal

Our general goal is to evaluate the feasibility and potential effectiveness of an HIV/CVD polypill in HIV-infected patients with at least moderate CVD risk, by studying candidate drug components (pravastatin and lisinopril) in patients receiving effective treatment with ART that do not require treatment with these medications by current recommendations. In this pilot trial, each participant will be randomized to receive pravastatin 20mg daily versus placebo <u>and</u> lisinopril 10mg daily versus placebo, and followed for 4 months.

1.2. Study Hypotheses

A. Adherence and tolerability to pravastatin (20mg) and lisinopril (10mg), given alone or in combination, will be high in this study population who are, by definition, engaged in care and adherent to their current ART regimens.
B. Framingham 10 year risk score (FRS) will improve with treatment of either pravastatin or lisinopril due primarily to the effect on blood lipids or BP, respectively.
C. The potential efficacy of pravastatin (20mg) and lisinopril (10mg), given alone or in combination, will also be assessed via several secondary outcomes: Biomarkers of inflammation, endothelial injury, coagulation, apolipoproteins, lipoprotein particle concentration and large and small artery elasticity (LAE and SAE).

1.3. HIV/ART-related CVD Risk

Concurrent with the advent of effective combination ART and a reduction in AIDS events, CVD has become a major cause of morbidity and mortality among HIV-infected patients.[22-24] Traditional risk factor assessments such as Framingham criteria remain informative for CVD risk among ART-treated HIV-infected patients, but do not incorporate additional risk related to viral replication or ART drug toxicity.[12 25] Longitudinal cohort data has shown that CVD event risk increases with each additional year of protease inhibitor (PI)-based ART, after adjusting for additional factors including lipids.[23] This pilot study will focus on treated HIV-infected participants with moderate CVD risk, assessed from Framingham risk score cutoffs and incorporating duration of PI exposure.

In the general population, mechanisms that promote atherosclerosis, in part, involve inflammation, thrombogenesis, adverse lipid and metabolic changes, and vascular damage and dysfunction.[26 27] These same factors are amplified among individuals infected with HIV, whether related to viral replication, ART use, or the increased prevalence of smoking.[2 4 23 28 29]

HIV infection and ART each have a substantial impact on blood lipids.[30-33] Among men in the Multicenter AIDS Cohort Study (MACS), HDLc, and LDLc decrease following HIV seroconversion.[33] Triglyceride levels (TG) are elevated with HIV infection, when compared to HIV-negative cohorts.[30 33 34] Starting ART is associated with a typical metabolic pattern including hypertriglyceridemia, hypercholesterolemia, lipodystrophy and alterations of glucose metabolism.[9 23] Specifically, ART initiation among HIV-infected patients in MACS led to increases in total cholesterol (TC) and LDLc above pre-infection levels, while HDLc remained approximately 10mg/dl below pre-infection levels.[33]

Strong evidence linking HIV- and ART-related increases in inflammation and thrombogenesis, and subsequent CVD risk, has come from recent studies of plasma specimens from SMART. Higher baseline levels of IL-6 and D-dimer were strongly associated with an increase mortality risk (IL-6 OR = 8.3, 3.3-20.8; D-dimer OR = 12.4, 4.2-37.0), and associations persisted for both CVD and non-CVD death.[35] A follow-up comparison of baseline biomarker levels in SMART participants with general population cohorts, including the Coronary Artery Risk Development in Young Adults (CARDIA) study and the Multi-Ethnic Study of Atherosclerosis (MESA), and found that hsCRP, IL-6 and D-dimer levels were 50-100% higher in HIV-infected participants both on and off ART ($p<0.001$ for all).[36] Thus, pro-inflammatory mechanisms persist in persons with HIV infection event after starting ART.

Endothelial function in persons with HIV infection may be a consequence of inflammation, HIV replication itself, and/or ART drug toxicity.[29 39-41] Endothelial cell adhesion molecules are elevated in persons with HIV infection, when compared to HIV negative controls.[42] Brachial artery flow mediated dilation (FMD) was impaired among 75 HIV infected participants when compared to an historic cohort of 223 HIV negative controls, and was associated with a detectable HIV RNA level.[3] We have also demonstrated that LAE and SAE is impaired among persons with untreated HIV infection compared to HIV-negative controls, after adjusting for additional risk factors including Framingham risk score.[43] Treatment with lisinopril and pravastatin in this proposal may counter mechanisms related to inflammation and vascular dysfunction.[20 21 46-48]

1.4. Blood Pressure and Lipid Lowering Treatment to Reduce CVD Risk

High BP and cholesterol cause more than half of all CVD globally.[49] Data from over 350,000 men part of the Multiple Risk Factor Intervention Trial (MRFIT) demonstrated a strong continuous CVD risk gradient with SBP and TC levels that extends below diagnostic thresholds for 'hypertension' or 'hypercholesterolemia', and risk across the board is amplified by smoking.[15] Data from large trials have confirmed the benefits of BP and lipid lowering treatment, with ACEi and statin drugs respectively, in moderate to high-risk patients who do not have 'hypertension' or 'hypercholesterolemia'.[14 50-52]

Recent findings from the Justification for the Use of Statins in Prevention: an Intervention Trial Evaluating Rosuvastatin (JUPITER) demonstrate that pre-emptive treatment with a 'statin' (HMG-CoA reductase inhibitor) medication reduced CVD events among persons with elevated hsCRP levels.[17-19] In JUPITER, a 50% reduction in CVD events was reported among 17,802 participants with elevated hsCRP levels (≥2mg/L) and LDLc <130mg/dL given a Rosuvastatin medication.[18] Whether a result of the 50% reduction in LDLc or the 37% reduction in hsCRP levels, the clinical benefit of pre-emptive statin treatment in this population with moderate CVD risk has direct implications for prevention strategies among HIV-infected persons. In addition, a recent study of 76 patients with a BP <140/90 mmHg, treatment with an angiotensin receptor blocker led to a 30% improvement in small artery elasticity, as well as lowering BP.[47] The use of ACEi or statin medications as primary prevention specifically among HIV-infected patients without 'hypertension' or 'hypercholesterolemia' has not been reported.

2. METHODOLOGY

2.1. Study Design

This proposal will study the tolerability and potential efficacy of pravastatin 20mg once daily and lisinopril 10mg once daily to reduce CVD risk among at least 40 HIV-infected patients receiving effective ART. A double blind, randomized, 2x2 factorial design will be utilized to compare each study with a matched placebo pill. After screening, 3 study visits are planned: baseline, month1 and month 4.

Study Population

Inclusion Criteria:

1. HIV-infected participants receiving ART with HIV RNA level <400 copies/mL, and
2. Framingham 10yr risk score ≥3%

Exclusion Criteria:

1) Known CVD or CVD equivalent (including DM or Framingham 10yr risk score >20%)
2) Unable to obtain a pulse waveform measure (e.g. due to atrial fibrillation)
3) Currently taking a statin, ACEi or antiotensin receptor blocker
4) BP ≥140/90mmHg at screening visit (the lowest of 3 values may be used)

HIV-infected Participants Taking Effective ART (n=40)

Randomized

	**Lisinopril 10mg daily** vs. **Placebo daily**		**Totals**
	Lisinopril 10mg daily	Placebo daily	
**Pravastatin 20mg daily**	Lisinopril + Pravastatin (n=10)	Placebo + Pravastatin (n=10)	20
vs.			
**Placebo daily**	Lisinpril + Placebo (n=10)	Placebo + Placebo (n=10)	20
**Totals**	20	20	**40**

5) LDL cholesterol ≥160mg/dL with Framingham 10yr risk score <10%
6) LDL cholesterol ≥130mg/dL with Framingham 10yr risk score 10-20%
7) TG >500mg/dL (must first treat to <500mg/dL)
8) History of cirrhosis or current ALT/AST ≥ 5 x the upper limit of normal
9) Chronic kidney disease with a creatinine ≥2.0 mg/dL
10) Contra-indications to any of the study drugs (or an angiotensin receptor blocker)

Intervention:
All participants will be randomized to take pravastatin 20mg daily or matched placebo, and lisinopril 10mg daily or a matched placebo, for 4 months. This is summarized in figure above.

2.2. Outcomes

Primary Outcomes
a. Medication tolerability
    i. Study withdrawal due to medication side effect (subjective or lab criteria)
    ii. Discontinuation of a single study medication due to side effect (subjective or lab criteria)
    iii. Lab measure with >2 fold increase (with emphasis on: Creatinine, CK, AST, ALT)
b. Adherence to study medication
    i. Self report of misses per week
    ii. Pill count by study RN – reported as % (pills taken/days enrolled in study)
c. Changes in Framingham risk score (FRS), including serum cholesterol levels and blood pressure

Secondary Outcomes
a. Changes biomarkers (TBD)
a. Changes in small artery elasticity (SAE)

2.3. Design Justification

General Design:
A randomized trial design will control for both known and unknown confounders, and the 2X2 factorial design will allow separate evaluation of the treatment interventions. Visit frequency was chosen to evaluate short-term tolerability and risk factor changes in an efficient manner with this initial pilot phase. Other CVD risk factors, such as smoking, will not specifically be targeted in the context of study visits so as not to confound the effect of pravastatin and/or lisinopril.

Targeted Study Population:
The purpose of this study is to study two components of a polypill (statin and ACEi) among HIV-infected participants who are at least at moderate CVD risk by traditional risk factor assessment. Both HIV-infection and ART increase CVD risk further, so the Framingham risk score cut-off for inclusion was low-moderate (see below). Primary prevention for CVD is more appropriate for patients at low risk for AIDS events, and, thus, we included only HIV-infected participants receiving effective ART.

CVD Risk Threshold (use of Framingham Risk Score):
Traditional CVD risk algorithms, such as Framingham, remain generally effective at predicting risk among HIV-infected patients.[25] The National Cholesterol Education Program Adult Treatement Panel III (ATP III)

designates CVD risk, based on traditional risk factors (smoking, BP≥140/90, HDLc <40mg/dL, family history of premature CVD and age ≥45 years for men and ≥55 years for women) and Framingham 10 year risk score (FRS), into 4 categories: 1) Lower risk is 0-1 risk factor and FRS <10; 2) Moderate risk is 2+ risk factors and FRS <10; 3) Moderately high risk is 2+ risk factors and FRS of 10-20; and 4) High risk is CVD or CVD equivalent, which may include a FRS >20.

We attempted to targeted HIV infected patients of at least moderate CVD risk, as the very lowest risk patients are less likely to benefit from primary prevention and BP and/or lipid-lowering therapy. Use of Framingham risk score for inclusion criteria allows consideration of age, gender, systolic BP, TC, HDLc and smoking status. ART exposure and HIV infection could also both be considered to increase CVD risk (see background). A Framingham risk score cutoff of ≥3% eliminates the participants at very low risk for CVD. Most recent estimates from the D:A:D (Data Collection on Adverse Events of Anti-HIV Drugs) study indicate risk for myocardial infarction increases 16% with each additional year of ART exposure. Applying these data, an HIV-infected patient with approximately 4 years of ART exposure, and a Framingham risk score of 3%, will have 10-year risk of CVD event of approximately 5%. This estimate does not account for increased risk of CVD associated with HIV infection itself. The use of a the Framingham 10-year risk score as a screening tool for this study was also motivated by the ease of implementing this strategy in clinical practice.

Study Drug:
The choice of drugs for this study was motivated by their efficacy as secondary prevention for CVD events, adjunct anti-inflammatory properties, availability as generic preparations, and potential to counter HIV- and ART-specific CVD risk mechanisms beyond BP and lipid lowering properties.[17-20 54] In addition, some statin medications are contraindicated when taking PIs (due to interactions with CYP3A4 pathway), and for this reason pravastatin is the preferred given a lack of metabolism through the CYP3A4 pathway.[53 55] Darunavir has been noted to increase pravastatin levels to a greater extent than other PIs, and participants taking darunavir will be counseled specifically about this but will not be excluded from participating for this reason.

Both pravastatin and lisinopril are available in generic form. These drugs also have a long track record of being used safely in a clinical setting. Participants for whom BP or cholesterol treatment is indicated will not be enrolled. In addition to clinical monitoring for side effects, toxicity labs will be included at study visit. A clinical doctor of pharmacy (PharmD) is included on the protocol team and will review each participant's medication list upon enrollment. If toxicity to study drugs develop, then participants will be un-blinded and discontinue any active study medication per monitoring criteria below.

2.4. Data Collection

Details of study will be reviewed with all interested participants. If inclusion criteria are likely based on review of clinic records then a screening visit may be scheduled, where labs and BP will be assessed. Informed consent will be obtained at screening visits. Screening blood draw results will be used to verify blood lipid eligibility criteria (LDLc and TG). The lowest BP reading, taken from up to 3 readings during a screening visit, may be used to assess eligibility criteria. Participants will be given an information pamphlet on ways to reduce heart disease risk at enrollment. If exclusion or withdrawal criteria are met due to BP or lipid level thresholds, then a note with these results will be sent to the participants primary care giver with recommendations that they be evaluated for hypertension or dyslipidemia, respectively.

If eligibility criteria are met, participants will be randomized at the baseline visit to occur within 2 months of screening/consent. After enrollment and baseline visit, participants will present month 1 and month 4 for repeat study procedures. The visit schedule is outlined in the table below. Visits will last approximately 60 minutes.

Toxicity labs and a clinical assessment will be scheduled at any point during the 4 months follow-up if new symptoms develop that may be related to study medications. Toxcitity labs and adherence will otherwise be assessed along with study procedures at scheduled 1 and 4 month visits.

TABLE: Study procedures and visit timeline

**Measure**	**Screening visit**	**Baseline visit**	**Symptoms?**	**Month 1 visit**	**Month 4 visit**
Informed consent	X				
Randomization		X			
BP	X	X		X	X
Lipids (TC, HDLc, LDLc, TG)	X	X (if not at screen)		X	X
FRS	X	X		X	X
Med Adherence			X	X	X
Hep B/C serologies	X	X (if not at screen)			
Toxicity labs (CMP, CBC, CK)	X	X (if not at screen)	X	X	X
HIV labs (RNA, CD4)	X (if not ≤3 months)	X (if not at screen)		X	X
Artery Elasticity		X		X	X
Biomarkers		X		X	X
Stored Specimens		X		X	X

Randomization will be predetermined using block-randomization with varied groups of 4 and 8, and blinded to investigators, staff and participants. An un-blinded statistician, one research staff not involved with study visits, and an HCMC pharmacist will have access to the randomization code.

Pravastatin and lisinopril will be purchased through HCMC pharmacy at reduced generic pricing. Active study drugs and placebo pills will be encapsulated (Custom-Rx Compounding Pharmacy in Richfield, MN) and then packaged and labeled with a study identification number. Study medications will be stored and dispensed by study investigators. Investigators will comply with HCMC policies on use of medications for ongoing IRB approved studies.

2.5. Sample Size Considerations

With a total sample size of 40 patients (20 per group for comparison of each drug intervention), a 0.9*SD (standard deviation) difference in 10-year Framingham risk score from each drug can be detected with 80% power at the 0.05 (2-sided) level of significance. The variability in Framingham risk score was estimated from the Multiple Risk Factor Intervention Trial (MRFIT), where the estimated SD based on 11,702 participants was 0.20, corresponding to a relative change of approximately 20%. Thus, a 0.9*SD change in this pilot study would correspond to an 18% relative change in Framingham risk score over the follow up period. Thus a change in Framingham risk of 8% to 6.5% would be >18% relative improvement. In this proposal, if a 40-year old male smoker with SPB of 130mmHg, TC of 190mg/dL,

HDLc 35mg/dL demonstrated a reduction in SBP of -5mmHg and TC of -20mg/dL, and no increase in HDLc, his 10-year Framingham risk score would have a 25% relative improvement (8% to 5%). This is a very reasonable estimate as a mean change in TC of -40mg/dL after 12 weeks of pravastatin treatment was reported among 86 HIV-infected participants, and patients in our HCMC clinic commonly have lipid profiles less favorable than this.[43]

2.6. Study procedures

At study visits participants will be assessed for study medication tolerability and toxicity, undergo a peripheral blood draw, and BP and artery elasticity measurements. Demographic characteristics, past medical history and additional risk factors will also be ascertained. Framingham 10-year risk score will be calculated using an NHLBI online algorithm **(http://hp2010.nhlbihin.net/atpiii/calculator.asp),** after entering age, gender, smoking status, TC, HDLc, systolic BP and use of a BP lowering medication.

Medication tolerability: At each visit, subjective symptoms will be assessed along with toxicity labs will be assessed at each visit. Clinical assessment and toxicity labs will also be measured outside the context of study visits if participants develop subjective side effects that may be related to study medications. Toxicity labs include:

i. Complete blood count (CBC)
ii. Complete metabolic panel (CMP: including liver function tests)
iii. Creatine phosphokinase (CK).

Adherence: Adherence will be assessed via participant report and pill count (by study RN) during follow-up visits.

Arterial Elasticity Measurements: Arterial elasticity will be assessed via pulse waveform analysis (model PulseWave CR-2000, Hypertension Diagnostics, Inc., Eagan, MN), which provides the following measurements: large and small artery elasticity index (ml/mmHg; LAE and SAE respectively), systemic vascular resistance (dyne•sec•cm^{-5}), total vascular impedance (dyne•sec•cm^{-5}), cardiac output (L/min), pulse rate (beats/min), and BP (mmHg). Measures will be assessed in triplicate.

Procedures for obtaining artery waveform measurements are described in the device operator's manual. Participants will be instructed to fast and avoid caffeine, nicotine, anti-histamines and NSAIDS prior to study visits.

Blood pressure: Blood pressure will be assessed during artery elasticity waveform measures, and estimates will also be ascertained in triplicate.

Laboratory Tests: At each study visit, a peripheral blood draw (<50ml) will be performed by a study investigator or RN. Samples will be de-identified and labeled with a study ID number. In addition to toxicity labs (above), HIV RNA level, CD4+ T cell count and a fasting lipid panel (total cholesterol, LDLc, HDLc, and triglycerides) will be measured through HCMC clinical lab. Serologies for Hepatitis B and C will also be measured at the baseline visit only. Blood tubes for clinical tests will be immediately sent to HCMC clinical labs for processing. The following specimens will be processed and stored for biomarker assays:

i. Citrated plasma: 10cc cyan blue

ii. Plasma (EDTA): 10cc lavender
iii. Plasma with heparin: 4cc green
iv. Serum: 4cc yellow
v. Platelet free plasma (CDAT): 4cc light-blue

Blood specimens for future biomarker analyses will be centrifuge blood at 2500 X g for 15 minutes (for a total of >30,000 g-minute spin) in a refrigerated centrifuge at 4°C. Before aliquoting, pre-label the 0.5cc storage vials. Using a transfer pipette and following aseptic techniques, immediately remove as much plasma as possible without disturbing the red cell layer. Transfer 0.5cc of plasma to each of the pre-labeled transport vials. Screw caps on tightly. Do not tape the caps on. Immediately freeze the vials in an upright position (at -80°C)

Citrated Plasma samples must be obtained without trauma (e.g. multiple sticks, patting venipuncture site, placing tourniquet on too tightly or for too long). This should be a Morning-Fasting (at least 8 hours, no food or drink except water) sample collected. Fill citrated plasma tube prior to obtaining any other labs, but after discarding a single tube with no additive (red-top). After collecting the participant's blood using 3.2% sodium citrate (light blue top) tube, gently invert the tube no more than 5 times to mix the blood and additive. Place filled citrate tube on wet ice and centrifuge within 30 minutes. Visually inspect sample. If a clot is present do not use the sample. The participant will need to have the sample re-drawn.

2.7. Recruitment and Enrollment

Participants will be primarily recruited from HCMC HIV clinic (Positive Care Center) through provider referral and informational flyers distributed in clinical exam rooms. Referrals will also be accepted from local providers in the Twin Cities. Research (‘Solutions’) and clinical (EPIC) databases will be queried for to identify eligible patients from our clinic that may be approached directly.

We anticipate that recruitment and enrollment through the HCMC HIV clinic will fill the study after 12-15 months. Recruitment progress will be assessed weekly with research staff. If recruitment is slow, we will encourage referrals from Park Nicollet ID clinic and Allina affiliated HIV clinics. Each of these clinics provides care for another 500-700 HIV-infected patients. In addition, protocol modification with expanded inclusion criteria may be considered after 6 months.

ADDENDUM – To enhance enrollment, arrangements were made in September 2010 to recruit participants and conducted study visits at the Allina HIV clinic (‘Clinic 42’) at Abbott Northwestern Hospital in Minneapolis.

3. CLINICAL MANAGEMENT

3.1. Training and Administration of Study Procedures

A single RN will be trained and perform all study visit procedures at each participating site. Study visits will take approximately 60 minutes. A lab technician will assist with blood specimen processing. Plasma/serum specimens will need to be processed within 30 minutes of collection. Artery waveform data will be transmitted from the HDI tonometer to a PC (as a text file), per instructions in the operator’s

manual, after each study visit. CRFs and artery waveform data will be entered into a FileMaker (v9) database.

3.2. Participant Safety Monitoring and Withdrawal

Participants will undergo clinical assessment for side effects to study medication at baseline and each follow-up visit (months 1 and 4). Toxicity labs will be assessed per 2.6 above at each study visit, or at any point during the study if symptoms suggestive of drug toxicity/intolerance develop. A clinical pharmacist will participate on protocol team (i.e., Kristen Gamache) and will review the active clinical medication list upon request for participants prior to enrollment – where potential drug-drug interactions will be identified. Participants taking darunavir (which may increase pravastatin levels to a greater extent that other PIs) will not be excluded from participating from the study, but if a trend is suspected toward more toxicity in these participants then darunavir use may be added to exclusion criteria. Participants may withdraw from the study at any time at their request, as described in the consent.

Participants will be un-blinded to study medication (and will stop any active drug) if toxicity is clinically suspected, or if any of the following criteria are met:

i. ALT/AST levels double from baseline, increase to ≥10 x upper limit of normal, or any elevation associated with clinical symptoms.
ii. CK increase to an absolute level of >4x upper limit of normal
iii. Serum creatinine increase >0.5 mg/dL <u>and</u> to an absolute level >2.0 mg/dL; if only one of these criteria are met toxicity labs will be repeated in 2 weeks.
iv. Blood pressure >140/90mmHg at baseline and month 1 (lowest value may be used at each visit), when a participant fulfilled the BP thresholds/requirements at screening visit
v. New clinical indication for ACEi or statin medication that was not present at enrollment

4. ANALYSIS PLAN

The general goal of this pilot study is to gather feasibility and efficacy data, utilizing a 2x2 factorial design (4 cells of n=10, n=40 total), to inform planning for a multi-center HIV/CVD polypill trial. We believe treatment with pravastatin (20mg daily) and lisinopril (10mg daily) will be well tolerated and be well adhered to by participants engaged in care and already adherent to ART. We hypothesize that Framingham risk score will improve with either drug intervention. Future sample size estimates will be generated from effect size related to changes in arterial elasticity and biomarkers.

Primary Analyses: Mean changes in FRS after 4 months will be studied with pravastatin use (versus placebo) and lisinopril use (versus placebo). The effect of both drugs combined, compared with double-placebo, will also be examined. Adherence and the frequency of intolerability, based on subjective or lab criteria, will also be compared between groups using the definitions listed in 'outcomes' (2.2). Elevations in lab values will also be categorized by severity (using established grades 1-4).

Secondary Analyses: Mean changes in hsCRP, IL-6, D-dimer, SAE, ApoB, ApoA1, and LDL and HDL particle concentrations will be studied after 4 months of pravastatin and lisinopril, given alone or in combination, using similar methods as above in primary analyses. Tests for correlation between change in these markers and BP, total cholesterol and FRS will also be considered. Regression models may be

considered to adjust for additional clinical characteristics (including, but not limited to traditional CVD risk factors), but power will be quite limited for these analyses.

REFERENCES:

1. Stein JH, Klein MA, Bellehumeur JL, McBride PE, Wiebe DA, Otvos JD, et al. Use of human immunodeficiency virus-1 protease inhibitors is associated with atherogenic lipoprotein changes and endothelial dysfunction. *Circulation* 2001;104(3):257-62.
2. El-Sadr WM, Lundgren JD, Neaton JD, Gordin F, Abrams D, Arduino RC, et al. CD4+ count-guided interruption of antiretroviral treatment. *N Engl J Med* 2006;355(22):2283-96.
3. Solages A, Vita JA, Thornton DJ, Murray J, Heeren T, Craven DE, et al. Endothelial function in HIV-infected persons. *Clin Infect Dis* 2006;42(9):1325-32.
4. Kuller LH, Tracy R, Belloso W, De Wit S, Drummond F, Lane HC, et al. Inflammatory and coagulation biomarkers and mortality in patients with HIV infection. *PLoS Med* 2008;5(10):e203.
5. Murphy R, Costagliola D. Increased cardiovascular risk in HIV infection: drugs, virus and immunity. *AIDS* 2008;22(13):1625-7.
6. Sabin CA, Worm SW, Weber R, Reiss P, El-Sadr W, Dabis F, et al. Use of nucleoside reverse transcriptase inhibitors and risk of myocardial infarction in HIV-infected patients enrolled in the D:A:D study: a multi-cohort collaboration. *Lancet* 2008;371(9622):1417-26.
7. Madden E, Lee G, Kotler DP, Wanke C, Lewis CE, Tracy RP, et al. Association of antiretroviral therapy with fibrinogen levels in HIV-infection. *AIDS* 2008;22:707-15.
8. The SMART/INSIGHT and D:A:D Study Groups. Use of nucleoside reverse transcriptase inhibitors and risk of myocardial infarction in HIV-infected patients enrolled in the SMART study. *AIDS* 2008;22:F17-F24.
9. Grinspoon S, Carr A. Cardiovascular risk and body-fat abnormalities in HIV-infected adults. *N Engl J Med* 2005;352(1):48-62.
10. Currier JS, Lundgren JD, Carr A, Klein D, Sabin CA, Sax PE, et al. Epidemiological evidence for cardiovascular disease in HIV-infected patients and relationship to highly active antiretroviral therapy. *Circulation* 2008;118(2):e29-35.
11. Dube MP, Lipshultz SE, Fichtenbaum CJ, Greenberg R, Schecter AD, Fisher SD. Effects of HIV infection and antiretroviral therapy on the heart and vasculature. *Circulation* 2008;118(2):e36-40.
12. Grinspoon SK, Grunfeld C, Kotler DP, Currier JS, Lundgren JD, Dube MP, et al. State of the science conference: Initiative to decrease cardiovascular risk and increase quality of care for patients living with HIV/AIDS: executive summary. *Circulation* 2008;118(2):198-210.
13. Grunfeld C, Kotler DP, Arnett DK, Falutz JM, Haffner SM, Hruz P, et al. Contribution of metabolic and anthropometric abnormalities to cardiovascular disease risk factors. *Circulation* 2008;118(2):e20-8.
14. Grundy SM, Cleeman JI, Merz CN, Brewer HB, Jr., Clark LT, Hunninghake DB, et al. Implications of recent clinical trials for the National Cholesterol Education Program Adult Treatment Panel III guidelines. *Circulation* 2004;110(2):227-39.
15. Neaton JD, Blackburn H, Jacobs D, Kuller L, Lee DJ, Sherwin R, et al. Serum cholesterol level and mortality findings for men screened in the Multiple Risk Factor Intervention Trial. Multiple Risk Factor Intervention Trial Research Group. *Arch Intern Med* 1992;152(7):1490-500.
16. Jackson R, Lawes CM, Bennett DA, Milne RJ, Rodgers A. Treatment with drugs to lower blood pressure and blood cholesterol based on an individual's absolute cardiovascular risk. *Lancet* 2005;365(9457):434-41.

17. Ridker PM, Cannon CP, Morrow D, Rifai N, Rose LM, McCabe CH, et al. C-reactive protein levels and outcomes after statin therapy. *N Engl J Med* 2005;352(1):20-8.
18. Ridker PM, Danielson E, Fonseca FA, Genest J, Gotto AM, Jr., Kastelein JJ, et al. Rosuvastatin to prevent vascular events in men and women with elevated C-reactive protein. *N Engl J Med* 2008;359(21):2195-207.
19. Palinski W, Tsimikas S. Immunomodulatory effects of statins: mechanisms and potential impact on arteriosclerosis. *J Am Soc Nephrol* 2002;13(6):1673-81.
20. Scholkens BA, Landgraf W. ACE inhibition and atherogenesis. *Can J Physiol Pharmacol* 2002;80(4):354-9.
21. Dandona P, Dhindsa S, Ghanim H, Chaudhuri A. Angiotensin II and inflammation: the effect of angiotensin-converting enzyme inhibition and angiotensin II receptor blockade. *J Hum Hypertens* 2007;21(1):20-7.
22. Friis-Moller N, Sabin CA, Weber R, d'Arminio Monforte A, El-Sadr WM, Reiss P, et al. Combination antiretroviral therapy and the risk of myocardial infarction. *N Engl J Med* 2003;349(21):1993-2003.
23. Friis-Moller N, Reiss P, Sabin CA, Weber R, Monforte A, El-Sadr W, et al. Class of antiretroviral drugs and the risk of myocardial infarction. *N Engl J Med* 2007;356(17):1723-35.
24. Lohse N, Hansen AB, Pedersen G, Kronborg G, Gerstoft J, Sorensen HT, et al. Survival of persons with and without HIV infection in Denmark, 1995-2005. *Ann Intern Med* 2007;146(2):87-95.
25. Schambelan M, Wilson PW, Yarasheski KE, Cade WT, Davila-Roman VG, D'Agostino RB, Sr., et al. Development of appropriate coronary heart disease risk prediction models in HIV-infected patients. *Circulation* 2008;118(2):e48-53.
26. Glasser SP, Selwyn AP, Ganz P. Atherosclerosis: risk factors and the vascular endothelium. *Am Heart J* 1996;131(2):379-84.
27. Ross R. The pathogenesis of atherosclerosis: a perspective for the 1990s. *Nature* 1993;362(6423):801-9.
28. Saves M, Chene G, Ducimetiere P, Leport C, Le Moal G, Amouyel P, et al. Risk Factors for coronoary heart disease in patients treated for human immunodeficiency virus infection compared with the general population. *CID* 2003;37:292-98.
29. Baker JV, Henry K, Neaton JD. The Consequences of HIV infection and Antiretroviral Therapy Use For Cardiovascular Disease Risk: Shifting Paradigms. *Current Opinion in HIV and AIDS* 2009;4:176-82.
30. Grunfeld C, Kotler DP, Hamadeh R, Tierney A, Wang J, Pierson RN. Hypertriglyceridemia in the acquired immunodeficiency syndrome. *Am J Med* 1989;86(1):27-31.
31. Grunfeld C, Pang M, Doerrler W, Shigenaga JK, Jensen P, Feingold KR. Lipids, lipoproteins, triglyceride clearance, and cytokines in human immunodeficiency virus infection and the acquired immunodeficiency syndrome. *J Clin Endocrinol Metab* 1992;74(5):1045-52.
32. Feingold KR, Krauss RM, Pang M, Doerrler W, Jensen P, Grunfeld C. The hypertriglyceridemia of acquired immunodeficiency syndrome is associated with an increased prevalence of low density lipoprotein subclass pattern B. *J Clin Endocrinol Metab* 1993;76(6):1423-7.
33. Riddler SA, Smit E, Cole SR, Li R, Chmiel JS, Dobs A, et al. Impact of HIV infection and HAART on serum lipids in men. *JAMA* 2003;289(22):2978-82.
34. Moyer MP, Tracy RP, Tracy PB, van't Veer C, Sparks CE, Mann KG. Plasma lipoproteins support prothrombinase and other procoagulant enzymatic complexes. *Arterioscler Thromb Vasc Biol* 1998;18:458-65.

35. Kuller LH, Tracy RP, Belloso W, DeWit S, Drumond F, Lane HC, et al. Activation of inflammatory and coagulation pathways is associated with mortality in patients with HIV infection. *PLoS Medicine* 2008;(in press).
36. Neuhaus for SMART study Investigators. Markers of Inflammation, Coagulation, and Renal Function in HIV-infected Adults in SMART and in Two Large Population-Based Stdies, CARDIA and MESA. 16th Conference on Retroviruses and Opportunistic Infections; 2009 February 8-11; Montreal, Canada.
37. Reingold J, Wanke C, Kotler D, Lewis C, Tracy R, Heymsfield S, et al. Association of HIV infection and HIV/HCV coinfection with C-reactive protein levels: the fat redistribution and metabolic change in HIV infection (FRAM) study. *J Acquir Immune Defic Syndr* 2008;48(2):142-8.
38. Sutherland JP, McKinley B, Eckel RH. The metabolic syndrome and inflammation. *Metab Syndr Relat Disord* 2004;2(2):82-104.
39. Ren Z, Yao Q, Chen C. HIV-1 envelope glycoprotein 120 increases intercellular adhesion molecule-1 expression by human endothelial cells. *Lab Invest* 2002;82(3):245-55.
40. Gross PL, Aird WC. The endothelium and thrombosis. *Seminars in Thrombosis and Hemostasis* 2000;26:463-78.
41. Bussolino F, Mitola S, Serini G, Barillari G, Ensoli B. Interactions between endothelial cells and HIV-1. *Int J Biochem Cell Biol* 2001;33(4):371-90.
42. de Gaetano Donati K, Rabagliati R, Iacoviello L, Cauda R. HIV infection, HAART, and endothelial adhesion molecules: current perspectives. *Lancet Infect Dis* 2004;4(4):213-22.
43. Baker JV, Duprez D, Rapkin J, Huppler Hullsiek K, H. Q, Grimm R, et al. Untreated HIV Infection and Large and Small Artery Elasticity. *JAIDS* 2009;*In press*.
44. Wolf K, Tsakiris DA, Weber R, Erb P, Battegay M. Antiretroviral therapy reduces markers of endothelial and coagulation activation in patients infected with human immunodeficiency virus type 1. *J Infect Dis* 2002;185(4):456-62.
45. Torriani FJ, Komarow L, Parker RA, Cotter BR, Currier JS, Dube MP, et al. Endothelial function in human immunodeficiency virus-infected antiretroviral-naive subjects before and after starting potent antiretroviral therapy: The ACTG (AIDS Clinical Trials Group) Study 5152s. *J Am Coll Cardiol* 2008;52(7):569-76.
46. Leibovitz E, Hazanov N, Zimlichman R, Shargorodsky M, Gavish D. Treatment with atorvastatin improves small artery compliance in patients with severe hypercholesterolemia. *Am J Hypertens* 2001;14(11 Pt 1):1096-8.
47. Duprez DA, Florea ND, Jones K, Cohn JN. Beneficial effects of valsartan in asymptomatic individuals with vascular or cardiac abnormalities: the DETECTIV Pilot Study. *J Am Coll Cardiol* 2007;50(9):835-9.
48. Akgullu C, Ozdemir B, Yilmaz Y, Kazazoglu AR, Aydinlar A. Effect of intensive statin therapy on arterial elasticity in patients with coronary artery disease. *Acta Cardiol* 2008;63(4):467-71.
49. The World Health Report 2002. Reducing risks, promoting healthy life. Geneva: World Health Organization, 2002.
50. Yusuf S, Sleight P, Pogue J, Bosch J, Davies R, Dagenais G. Effects of an angiotensin-converting-enzyme inhibitor, ramipril, on cardiovascular events in high-risk patients. The Heart Outcomes Prevention Evaluation Study Investigators. *N Engl J Med* 2000;342(3):145-53.
51. Randomised trial of a perindopril-based blood-pressure-lowering regimen among 6,105 individuals with previous stroke or transient ischaemic attack. *Lancet* 2001;358(9287):1033-41.
52. MRC/BHF Heart Protection Study of cholesterol lowering with simvastatin in 20,536 high-risk individuals: a randomised placebo-controlled trial. *Lancet* 2002;360(9326):7-22.

53. Dube MP, Stein JH, Aberg JA, Fichtenbaum CJ, Gerber JG, Tashima KT, et al. Guidelines for the evaluation and management of dyslipidemia in human immunodeficiency virus (HIV)-infected adults receiving antiretroviral therapy: recommendations of the HIV Medical Association of the Infectious Disease Society of America and the Adult AIDS Clinical Trials Group. *Clin Infect Dis* 2003;37(5):613-27.
54. Cohn JN. What is the role of angiotensin-receptor blockade in cardiovascular protection? *Am Heart J* 2006;152(5):859 e1-8.
55. Fichtenbaum CJ, Gerber JG, Rosenkranz SL, Segal Y, Aberg JA, Blaschke T, et al. Pharmacokinetic interactions between protease inhibitors and statins in HIV seronegative volunteers: ACTG Study A5047. *AIDS* 2002;16(4):569-77.
56. Aberg JA, Zackin RA, Brobst SW, Evans SR, Alston BL, Henry WK, et al. A randomized trial of the efficacy and safety of fenofibrate versus pravastatin in HIV-infected subjects with lipid abnormalities: AIDS Clinical Trials Group Study 5087. *AIDS Res Hum Retroviruses* 2005;21(9):757-67.

CARDIOVASCULAR RISK REDUCTION FOR PERSONS WITH HIV INFECTION: *a polypill pilot study*

Funded by the American Heart Association (AHA)

Short Title of the Study: AHA pilot

CONSENT FOR PARTICIPATING IN AN AHA-FUNDED RESEARCH TRIAL

SITE LEADER: *[insert primary site investigator]* **PHONE: *[insert phone]***

INTRODUCTION AND PURPOSE: WHY IS THIS STUDY BEING DONE?
Heart disease, or cardiovascular disease, is now a leading cause of illness and premature death among persons with HIV-infection. Safe, effective, and inexpensive treatment strategies that reduce risk for heart disease specifically for persons with HIV infection are needed.

The purpose of this study is to test the feasibility and tolerability of giving low-doses of a blood pressure medication (lisinopril) and a cholesterol medication (pravastatin) for reducing heart disease risk. Our long-term goal is to combine low doses of several medications into a single pill with minimize side effects that will prevent heart disease among persons with HIV infection.

YOUR PARTICIPATION IS VOLUNTARY
This consent form gives you information about the clinical research study that will be discussed with you. Once you understand the study, and if you agree to take part, you will be asked to sign this consent form. You will be given a copy to keep.

Before you learn about the study, it is important that you know the following:

- Your participation is entirely voluntary;
- You may decide not to take part or to withdraw from the study at any time without losing the benefits of your routine medical care.

Eligibility: Who is being asked to be part of this research study?
You are eligible for this study because you are over age 40, you have HIV infection and you are doing well with taking your antiretroviral medications. Specifically your HIV 'viral load' must be undetectable and you must not be taking, or have an indication to take, lisinopril, pravastatin, or similar medications. If your doctor or the study investigators feel that it would not be safe to take the study medications then you will not be eligible to participate.

We will also examine your risk for heart disease using an algorithm called the 'Framingham Risk Score', which predicts your risk for having a heart attack in the next 10 years. If this is too high,

we will recommend that you and your doctor consider starting medications for heart disease prevention and you will not be eligible for the study. Finally, if we are unable to perform any of the study procedures you may not be eligible for the study.

HOW LONG WILL YOU BE IN THE STUDY?
If you qualify for this study and sign this consent after your screening visit, you will be enrolled in the study. The study will then last approximately 4 months. You will come in for a 'baseline' study visit, and then a follow-up visit at 1 and 4 months.

HOW WILL THE STUDY WORK?
If you agree to participate in this study, you will be asked to take 2 study pills daily for 4 months. One pill will be either lisinopril or a placebo medication ('sugar pill'), and the other pill with be either pravastatin or a placebo medication. We will determine by chance whether your pill will be an 'active' of placebo medication, and neither you nor the study investigators will know which of these you will be taking. For each of the 2 pills, you have equal chance of being assigned an active medication or a placebo medication. You will start these study pills after the 'baseline' study visit, and will return for a 1 month and 4 month visit.

PROCEDURES: WHAT DO YOU HAVE TO DO IF YOU ARE IN THIS STUDY?

Screening visit:
You will be asked to come in for a screening visit, where you study investigators will review the study procedures and this consent form. If you agree to participate, you will have your blood drawn and medical history reviewed (similar to a clinic visit) to determine if you are eligible to participate. If you meet study criteria, you will then return within 1 month to begin the study.

Baseline and Follow-up visits:
Each visit will last approximately one hour, and will consist of:

1) Medical History: You will be interviewed to review your medical history and assess your risk for developing heart disease. We will also access your medical chart to obtain the results of recent lab tests related to your HIV status and heart disease risk.

2) Artery Pulse Waveform Analysis: We will record your pulse waveform measured at your wrist for a brief period while you are resting (using a hand held device: HDI/CR-2000). This involves no more discomfort that getting your blood pressure checked. This device estimates how well your vessels are functioning, including the ability of your vessels to relax. Your blood pressure, height and weight will also be measured.

3) Blood Draw – We will obtain a blood sample (approximately 8-10 tablespoons) from a vein in your arm. Blood samples will be used to measure markers in the blood related to heart disease and other complications of HIV. These samples may be stored for up to 20 years.

HOW WILL YOU GET MEDICINES FOR THE STUDY?
Study medications will be provided to you by the study and will be distributed by study nurses at the baseline and month 1 study visit. You will have sufficient supply to last the duration of the 4-month study. You will be asked to return all study medication, including empty containers, at the final study visit.

WHAT ARE THE RISKS AND/OR DISCOMFORTS OF THIS STUDY?

Possible risks
Your study pills may or may not contain an 'active' drug. Specifically, you have a 25% chance of taking 2 placebo pills during the study. The 'active' study medications (lisinopril and pravastatin) may cause side effects and you will be monitored for these at each visit. Lisinopril can cause a cough, or 'swelling' around the mouth or tongue, or a decrease in kidney function. If any of these occur you may stop this medication or withdraw from the study all together. Pravastatin can cause muscle aches or a decrease in liver function. If any of these occur you may stop this medication or withdraw from the study all together.

Both pravastatin and lisinopril are available in generic form. These drugs also have a long track record of being used safely in medical practice. The use of low doses of medications in this study may decrease the chance of side effects. You will also be monitored for side effects at each visit, and your lab tests will include an evaluation for signs of medication toxicity.

Risks of medicine interactions (where one medicine affects how another works)
A clinical doctor of pharmacy (PharmD) is participating in this study and will review your medication list upon enrollment for possible drug-drug interactions. Many 'statin' medications interact with HIV antiretroviral medications, but this occurs less often with pravastatin. Your HIV labs (including 'viral load') will be assessed at each study visit to be sure your HIV medications continue to work effectively during the study. At each study visit, the results of your laboratory tests will also help detect any other possible drug-drug interactions.

Risks of Study Procedures
There are no known risks associated with having your pulse waveform measured. You will have your blood drawn at each study visit. This is identical to having your blood drawn at a medical clinic, and can involve discomfort and/or minor bruising.

WHAT ABOUT PREGNANCY AND BREASTFEEDING?

If you are pregnant, breastfeeding or planning to become pregnant, you will not be eligible for this study. If you become pregnant during the study, you will be asked to withdraw. Therefore, if it is possible that you could become pregnant, we ask that you use at least 1 form of birth control (condoms, etc.) or abstain from sex during the 4 month study period.

WHAT ARE THE BENEFITS OF THIS STUDY?
If you take part in this study, there may be a direct benefit to you, but no guarantee can be made. It is also possible that you may receive no benefit from being in this study. What we learn from this study may help us prevent heart disease and improve the treatments of other people who are infected with HIV. By being in a research study, you may find out sooner about treatments, services, or other things that could help you live with your HIV infection than you would if you were not in a research study.

COMPENSATION
You will be paid $20 for each study visit you attend after the screening visit. You may receive a total of $60 for participating in the study. Study investigators may ask for your social security number as part of the monitoring process for this compensation.

WHAT IF THERE ARE NEW FINDINGS?
At the beginning of the study, you will be given general information about how to decrease your risk for heart disease. If your blood pressure readings or cholesterol levels are elevated during the study, the results will be given to you and sent to your primary provider to discuss management options with you. If these are consistently elevated, then you may be asked to withdraw from the study so that you may discuss treatment sooner with your primary provider. You will also be told about any new information learned during the study that might cause you to change your mind about staying in the study.

WHAT IF YOU DON'T WANT TO BE IN THE STUDY ANY LONGER?
If you enroll in this study, you may decide to stop participating at any time. Withdrawing from this study will not affect the benefits of your regular medical care.

CAN YOUR STUDY PARTICIPATION BE STOPPED WITHOUT YOUR CONSENT?
You may be taken off of study medicines before the end of the study if study investigators or your doctor recommend this. You may be taken off the entire study without your consent if:

- Your study doctor decides that continuing in the study would harm you;
- Your lab results indicate that you are experiencing toxicity from study medications;
- You doctor believes that your blood pressure or cholesterol should be treated more aggressively than is occurring in this study

WHAT OTHER CHOICES DO YOU HAVE BESIDES THIS STUDY?

You may discuss other strategies for reducing your risk for heart disease with your doctor. You will also be given general information about ways to decrease your risk for heart disease at the beginning of this study.

WHAT ARE THE COSTS TO YOU?
The medications that are part of this study will be provided free-of-cost to you, and will be distributed during study visits. During the study, you, your insurance company, or some other third-party payer must pay for all other medicines, including HIV medicines not paid by the study and medicines needed to prevent or treat other illnesses. We will provide all clinical and professional services, lab work, and other tests that are part of this study and not part of your regular care at no cost to you.

HOW IS YOUR PRIVACY PROTECTED?
Researchers will take every reasonable step to protect the privacy of your health information and to prevent misuse of this information. You will not be identified by name or any other way in any publication about this study. You will be identified only by a code, and personal information from your records will not be released without your written permission. Research staff will keep your files in a locked cabinet in a safe place and will handle your personal information very carefully. This will also help to protect your privacy.

Your medical and research records may be reviewed by the Hennepin County Medical Center ethics committee (institutional review board, IRB), and the research staff and monitors, and their designees. By signing this consent you are agreeing that your personal health information, and laboratory or other study results, may be shared with other study investigators, with research staff and with your HIV provider.

WHAT IF YOU ARE INJURED?
If you are injured as a result of being in this study, you will be given immediate treatment. The cost for treatment will be charged to you or your insurance company. There is no program through this institution or the American Heart Association to compensate participants who have research related injuries. You will not be giving up any of your legal rights by signing this consent.

WHAT IF YOU HAVE PROBLEMS OR QUESTIONS?
If you ever have questions about this study or in case of research-related injuries, you should contact ***[insert site investigator and phone]***. If you have questions about research subject's rights you can call ***[insert site IRB or Human Subjects Committee director with phone]***.

SIGNATURE PAGE FOR CONSENT TO PARTICIPATE IN THE STUDY OF 'CARDIOVASCULAR RISK REDUCTION FOR PERSONS WITH HIV INFECTION'

I have read this consent form, had the opportunity to ask questions and have received answers to any questions I have asked. I willingly give my consent to participate in this study, and authorize the use and disclosure of my health information as described in this form. By signing this consent form I do not give up any of my legal rights. Upon signing this form I will be given a signed copy of the form for my records.

If you have read the informed consent (or if you have had it explained to you) and understand the information, and you voluntarily agree to join this study, please sign your name below.

______________________<br>Participant's name<br>(typed or printed)	________________________<br>Participant's signature	__________<br>Date
	**OR**	
______________________<br>Participant's legal<br>guardian or representative	________________________<br>Legal guardian's signature	__________<br>Date

__________________________<br>Witness's name<br>(typed or printed)	______________________________<br>Witness's signature	_______<br>Date
